# Effects of CreER^T2^, 4-OH Tamoxifen, and Gender on CFU-F Assays

**DOI:** 10.1371/journal.pone.0148105

**Published:** 2016-02-01

**Authors:** Sophie L. McHaffie, Nicholas D. Hastie, You-Ying Chau

**Affiliations:** 1 MRC Human Genetics Unit, MRC Institute of Genetics and Molecular Medicine, University of Edinburgh, Edinburgh, United Kingdom; 2 University/BHF Centre for Cardiovascular Science, University of Edinburgh, Edinburgh, United Kingdom; The University of Adelaide, AUSTRALIA

## Abstract

Gene function in stem cell maintenance is often tested by inducing deletion via the Cre-*loxP* system. However, controls for Cre and other variables are frequently not included. Here we show that when cultured in the presence of 4-OH tamoxifen, bone and marrow cells containing the CreER^T2^ construct have a reduced colony forming ability. Inactive CreER^T2^ recombinase, however, has the opposite effect. Young female marrow cells containing the inactive CreER^T2^ construct grew more colonies than cells lacking the construct altogether. Young female control marrow cells (i.e., negative for CreER^T2^) also produced significantly greater colony numbers when cultured with 4-OH tamoxifen, compared with the ethanol vehicle control. In conclusion, we report that the use of the Cre-*loxP* system is inadvisable in combination with CFU-F assays, and that appropriate controls should be in place to extend the future use of Cre-*loxP* in alternate assays.

## Introduction

The colony forming unit-fibroblast (CFU-F) assay is frequently used to characterise stromal marrow cells and assess the number of mesenchymal progenitors [1–3]. These assays are performed using a population of flushed bone marrow in which the adherent stromal cells form colonies originating from a single cell; the CFU-F [2,3,4–6]. There are several other methods of obtaining material for CFU-F assays including the crushing of pre-flushed long bones followed by enzymatic digestion [3]. Both methods are used in this study.

Blood in human bone marrow has an oxygen level of ~7%, with mathematical models determining oxygen levels of ~1% to ~5% in the bone marrow, from the inner bone surface to the sinuses respectively [7–9]. It is therefore unsurprising that the low oxygen levels in the bone marrow have been found to extend the lifespan of mesenchymal stromal/stem cells (MSCs) and allow them to keep their stemness, i.e. proliferate without differentiating [10]. Compared to the marrow with its raised O_2_ level, the numbers of MSCs found are 4-fold higher on the surface of the trabecular bone, where the O_2_ levels are lower [10]. The MSCs have a significantly increased proliferative lifespan when cultured at 3% O_2_ *in vitro* and when cultured at 20% O_2_ have reduced stemness and undergo differentiation [10].

Typically, bone marrow studies (For example: [11,12]) are routinely carried out under standard culture incubator conditions of 5% CO_2_ and 95% air (20% O_2_). This does not reflect the physiological conditions for most mammalian tissues, as 3% O_2_ is a more appropriate condition for studying primary bone and marrow derived MSCs [10].

Therefore, this study was conducted at both normoxic (20% O_2_) and the more physiologically accurate “hypoxic” (3% O_2_) conditions. There are various gender differences found within stem cell groups, including MSCs, and so both sexes were also compared within this study [13].

Between 4 weeks and 12 weeks of age the murine femur length rapidly increases, after which growth of the long bones appears to stop [14]. By 12 weeks of age the number of stem cells in the murine bone and bone marrow has subsided [15]. Therefore to assess whether Cre is having an effect on adult cells only or also during earlier postnatal stages two ages groups were compared; adult mice (12–21 weeks old) and young mice (4 weeks old) which are still growing and therefore the skeleton is still developing.

Deletion via the Cre-*loxP* recombination method occurs when Cre recombinase causes the recombination of two 34bp *loxP* recognition sites [16]. It is frequently used for general and conditional gene knockouts plus reporter strains in studies carried out across an array of organisms e.g. animals [16–18], yeasts [19], and plants [20,21], but is known to have negative effects on cell cycle and proliferation rates [22]. In most cases the Cre recombinase is driven by a specific promoter resulting in targeted gene knockout [17,18]. In this case, CreER^T2^ is driven by the ubiquitous CAGG promoter in a tamoxifen dependent manner. Gene knockouts can consequently affect CFU-F assay outcomes, but often are not validated by important controls for Cre and other variables. Our results demonstrate the importance of Cre controls when using bone and marrow stromal cells for CFU-F assays.

## Results

### CreER^T2^ activation by 4-OH tamoxifen in adult bone and marrow cells reduces CFU-F colony numbers

**Fig 1:**
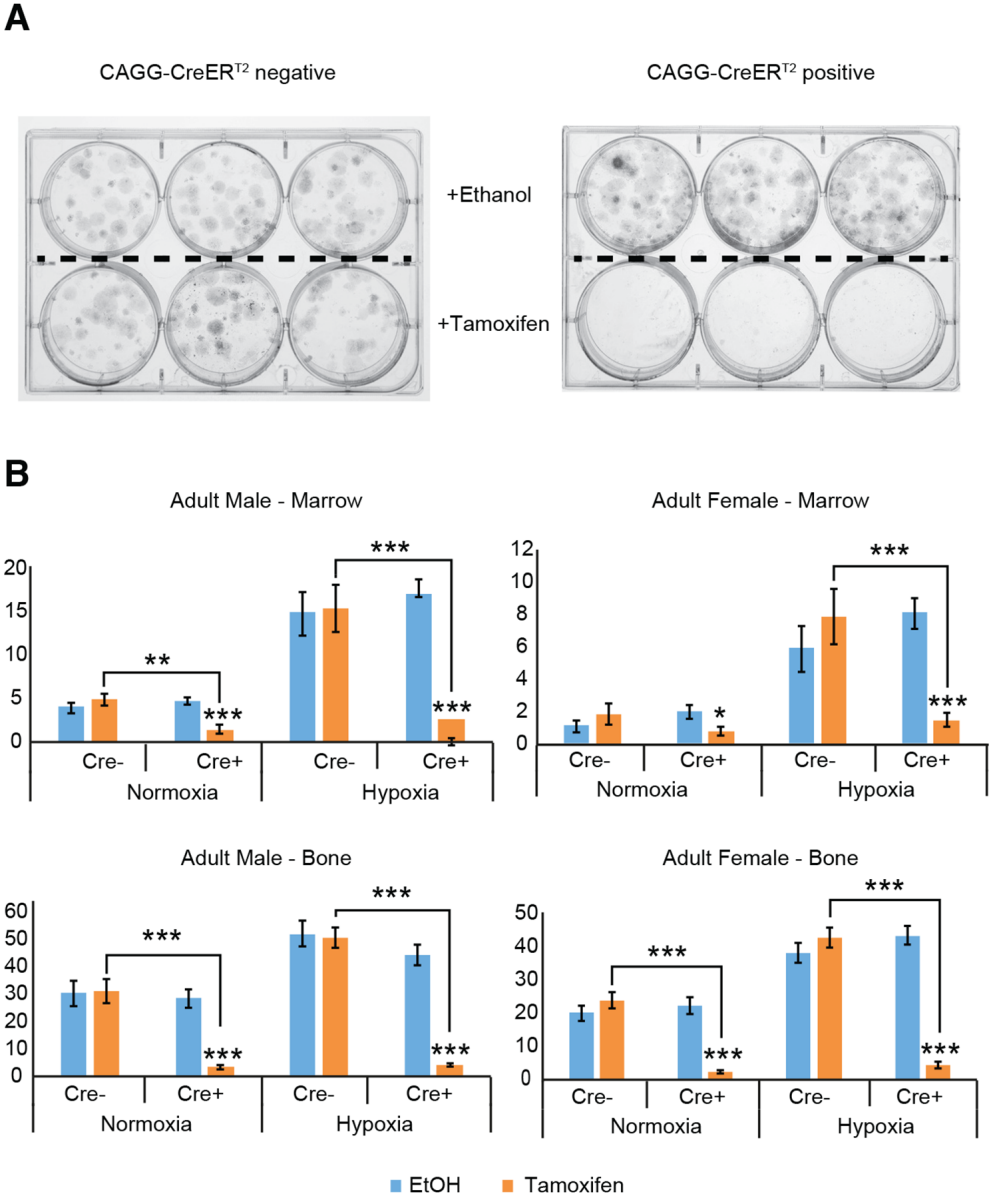
Colony forming abilities are reduced when adult CreER^T2^ positive cells are cultured with 4-OH tamoxifen. (A) Brightfield images of representative colonies from male bone cells cultured under hypoxia. (B) The colony forming assays were performed by culturing cells from central marrow and enzymatically digested flushed long bone for 10 days following culture in media with 4-OH tamoxifen (1μM) or vehicle control ethanol. Mean (±SEM) CFU-F assay colony numbers are reduced following CreER^T2^ activation with 4-OH tamoxifen in all Cre^+^ marrow and bone cells, irrespective of sex or culture conditions, compared to culture with ethanol. Mean (±SEM) CFU-F assay colony numbers are also reduced following CreER^T2^ activation with 4-OH tamoxifen in Cre^+^ male marrow and bone cells, female bone cells, and female hypoxic marrow, compared to Cre^-^ culture with 4-OH tamoxifen. *p<0.05 **p<0.01 ***p<0.001. (Cre^-^: n = 3 experiments. Cre^+^: n = 4 experiments. All experiments were performed in technical triplicate). (y axis = mean number of colonies with diameter greater than 1mm).

CreER^T2^ recombinases are generated by fusing Cre to the estrogen receptor (ER), rendering the CreER^T2^ recombinase inactive. It can then be activated by 4-OH tamoxifen; a synthetic ligand for the estrogen receptor [23]. CAGG-CreER^T2^ positive (Cre^+^) mice were compared against CAGG-CreER^T2^ negative (Cre^-^) mice to assess CreER^T2^ recombinase effects. CFU-F assays in the presence of 4-OH tamoxifen showed a significant decrease in colonies originating from Cre^+^ cells compared to Cre^-^ cells, as well as compared to Cre^+^ cells cultured with the vehicle control 100% ethanol (Representative colony images shown in Fig 1A). [Fig 1B: Cre^+^ vs. Cre^-^ (with tamoxifen): p<0.01 male normoxia marrow, p<0.001 male hypoxia marrow, male hypoxia and normoxia bone, female hypoxia marrow and female normoxia and hypoxia bone]. [Fig 1B: Cre^+^ (ethanol) vs. Cre^+^(tamoxifen): p<0.05 female normoxia marrow, p<0.001 all remaining comparisons].

### CreER^T2^ activation by 4-OH tamoxifen in adult bone and marrow cells reduces CFU-F colony numbers, irrespective of differentiation status

**Fig 2:**
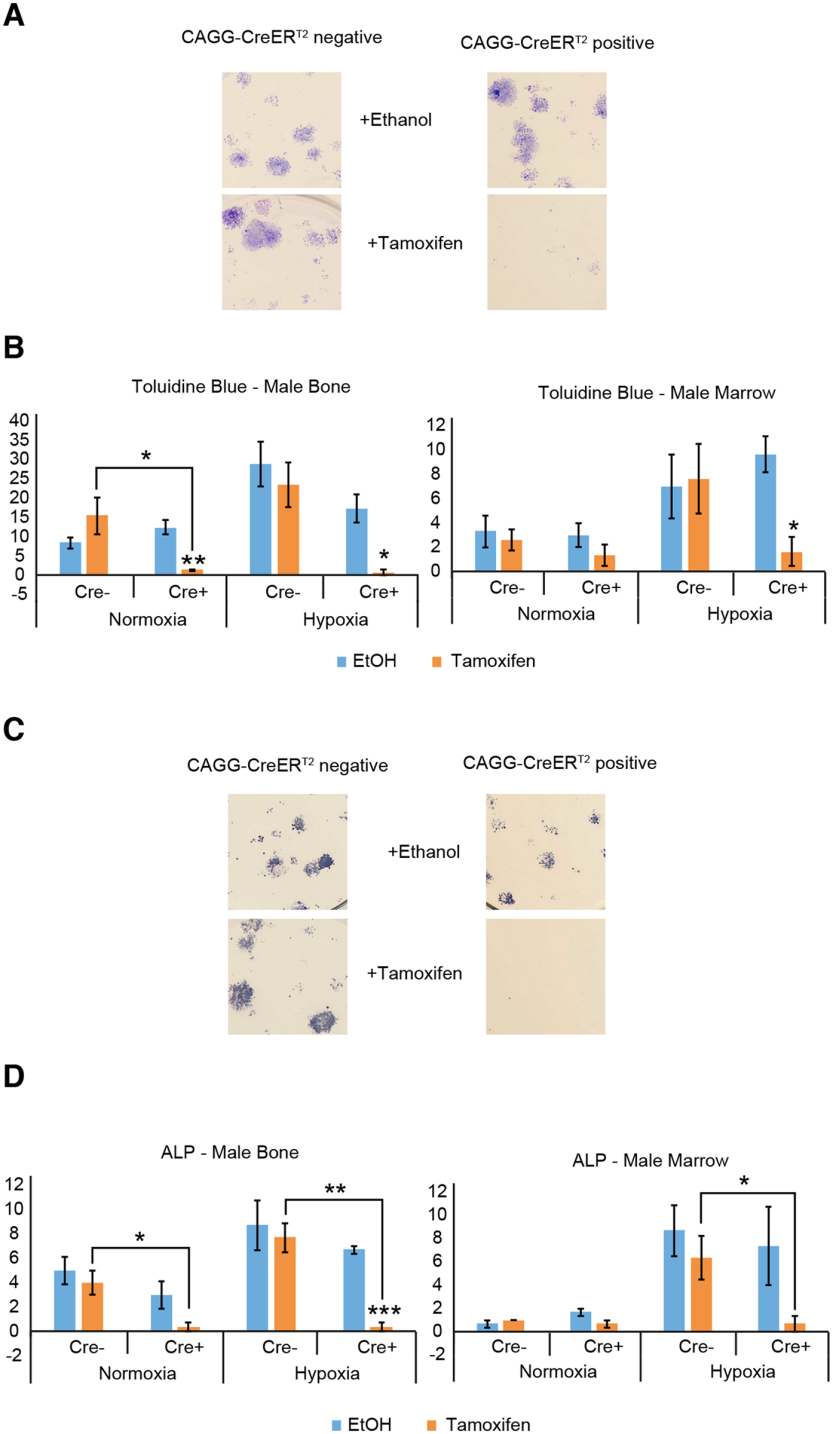
Colony forming abilities are reduced when adult CreER^T2^ positive cells are cultured with 4-OH tamoxifen. (A) Brightfield images of representative colonies stained with Toluidine Blue from male marrow cells cultured under hypoxia. (B) The colony forming assays were performed by culturing male cells from central marrow and enzymatically digested flushed long bone for 10 days following culture in media with 4-OH tamoxifen (1μM) or vehicle control ethanol. Colonies were then stained with Toluidine Blue to assess cartilaginous matrix content. Mean (±SEM) Toluidine Blue positive CFU-F assay colony numbers are reduced following CreER^T2^ activation with 4-OH tamoxifen in all Cre^+^ normoxic and hypoxic bone cells and hypoxic marrow cells, compared to culture with ethanol. Mean (±SEM) Toluidine Blue positive CFU-F assay colony numbers are also reduced following CreER^T2^ activation with 4-OH tamoxifen in Cre^+^ normoxic bone cells, compared with Cre^-^ cells cultured with 4-OH tamoxifen. (C) Brightfield images of representative colonies stained with Alkaline Phosphatase from male marrow cells cultured under hypoxia. (D) Colonies were then stained with ALP to assess the degree of bone formation. Mean (±SEM) ALP positive CFU-F assay colony numbers are reduced following CreER^T2^ activation with 4-OH tamoxifen in Cre^+^ hypoxic bone cells, compared to culture with ethanol. Mean (±SEM) ALP positive CFU-F assay colony numbers are also reduced following CreER^T2^ activation with 4-OH tamoxifen in Cre^+^ normoxic and hypoxic bone cells and hypoxic marrow cells, compared with Cre^-^ cells cultured with 4-OH tamoxifen. *p<0.05 **p<0.01 ***p<0.001. (Cre^-^: n = 3 experiments. Cre^+^: n = 4 experiments. All experiments were performed in technical triplicate). (y axis = mean number of colonies with diameter greater than 1mm).

Chondrogenesis is one of various processes identified by staining with toluidine blue. Colonies containing cartilage matrix will stain purple, while undifferentiated colonies will appear blue [24]. Bone alkaline phosphatase (ALP) activity is found in maturing chondrocytes, cartilage matrix, pre-osteoblasts, osteoblasts, osteocytes, and endosteal cells [25]. This expression profile means that ALP staining is useful for observing bone formation [26]. (Representative toluidine blue colony staining and ALP colony staining are shown in Fig 2A and 2C, respectively).

Not all colonies will be generated from pure stem cells, some may be from committed progenitors and so their level of stemness will be varied. Activated CreER^T2^ recombinase was seen to exert a negative effect on the number of both toluidine blue and ALP stained colonies (Fig 2), showing that this Cre effect occurred irrespective of differentiation status. This confirms and validates the CFU-F results by an alternative means. The proportion of colonies positive for ALP activity was not affected by CreER^T2^ recombinase presence, CreER^T2^ recombinase activation, ethanol presence or tamoxifen presence (Table A and B in S1 Table). [Fig 2B: Cre^+^ vs. Cre^-^ (with tamoxifen): p<0.05 male normoxia bone. [Fig 2B: Cre^+^ (ethanol) vs. Cre^+^ (tamoxifen): p<0.05 male hypoxia bone and marrow, p<0.01 male normoxia bone]. [Fig 2D: Cre^+^ vs. Cre^-^ (with tamoxifen): p<0.05 male normoxia bone, male hypoxia marrow, p<0.01 male hypoxia bone]. [Fig 2D: Cre^+^ (ethanol) vs. Cre^+^(tamoxifen): p<0.001 male hypoxia bone].

### CreER^T2^ activation by 4-OH tamoxifen in young bone and marrow cells reduces CFU-F colony numbers

**Fig 3:**
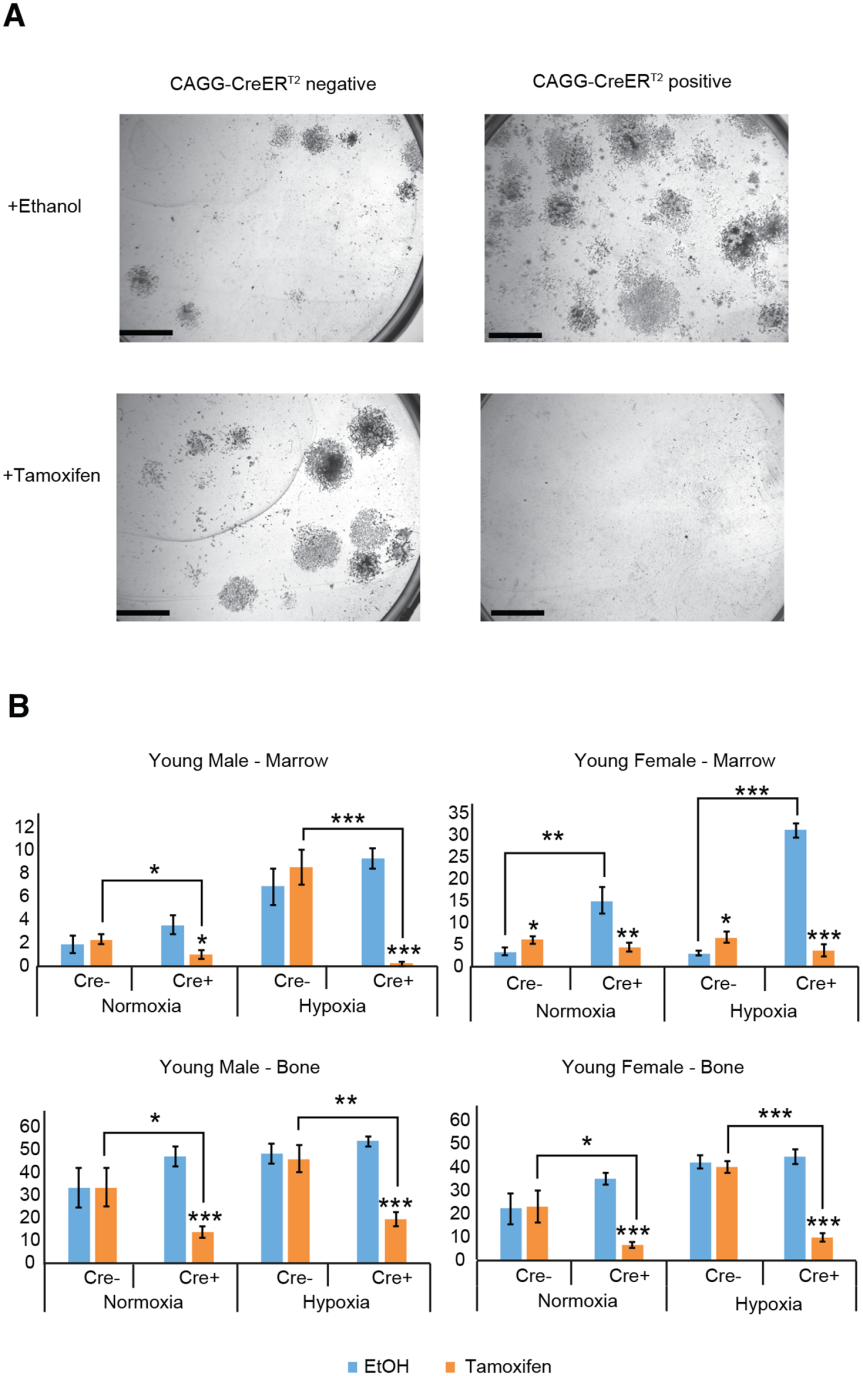
Colony forming abilities are reduced when CreER^T2^ positive cells from young mice are cultured with 4-OH tamoxifen. Young female CreER^T2^ positive marrow cells form more colonies than CreER^T2^ negative cells in the absense of 4-OH tamoxifen, and young female marrow CreER^T2^ negative cells cultured with 4-OH tamoxifen give rise to more colonies than when cultured with the ethanol vehicle control. (A) Brightfield images of representative colonies from female marrow cells cultured under hypoxia (scale bar = 5mm). (B) The colony forming assays were performed by culturing cells from central marrow and enzymatically digested flushed long bone for 10 days following culture in media with 4-OH tamoxifen (1μM) or vehicle control ethanol. Mean (±SEM) CFU-F assay colony numbers are reduced following CreER^T2^ activation with 4-OH tamoxifen in all Cre^+^ marrow and bone cells, irrespective of sex or culture conditions, compared to culture with ethanol. Mean (±SEM) CFU-F assay colony numbers are also reduced following CreER^T2^ activation with 4-OH tamoxifen in Cre^+^ male marrow and bone cells, and female bone cells, compared to Cre^-^ culture with 4-OH tamoxifen. Mean (±SEM) CFU-F assay colony numbers are higher from female Cre^+^ marrow cells cultured under both normoxia and hypoxia with ethanol, compared to Cre^-^ marrow cells with ethanol. Mean (±SEM) CFU-F assay colony numbers are increased in female Cre^-^ marrow cells cultured with 4-OH tamoxifen compared to ethanol, when cultured under both normoxia and hypoxia. *p<0.05 **p<0.01 ***p<0.001. (n = 3 experiments. All experiments were performed in technical triplicate). (y axis = mean number of colonies with diameter greater than 1mm).

CFU-F assays in the presence of 4-OH tamoxifen showed a significant decrease in colonies originating from Cre^+^ cells compared to Cre^-^ cells, as well as compared to Cre^+^ cells cultured with the vehicle control ethanol (Representative colony images shown in Fig 3A). [Fig 3B: Cre^+^ vs. Cre^-^ (with tamoxifen): p<0.05 male normoxia bone and marrow, female normoxia bone, p<0.01 male hypoxia bone, p<0.001 male hypoxia marrow and female hypoxia bone]. [Fig 3B: Cre^+^ (ethanol) vs. Cre^+^ (tamoxifen): p<0.05 male normoxia marrow, p<0.01 female normoxia marrow, p<0.001 male hypoxia marrow, female hypoxia marrow, male normoxia and hypoxia bone, and female normoxia and hypoxia bone].

### Inactive CreER^T2^ in young female marrow cells increases CFU-F colony numbers

100% ethanol was used as a vehicle for 4-OH tamoxifen and was included at the same concentration in culture media as a control. When Cre^+^ and Cre^-^ cells were cultured in this way a dramatic difference in female marrow colony numbers was observed (representative colony images shown in Fig 3A). Cre^+^ young female marrow cells had a significantly higher number of colonies compared to Cre^-^ cells (Fig 3B: p<0.01 normoxia, p<0.001 hypoxia), with the young male marrow and both female and male adult marrow cells showing a similar trend. As these cells were of pure genetic background and only cultured with media containing ethanol this suggests that the cause was the mere presence of inactive CreER^T2^ in the cytoplasm. This stimulatory effect is opposite to that seen when cultured with 4-OH tamoxifen and activated in the nucleus, suggesting that this is not a case of leaky Cre.

This study has also uncovered a fascinating gender dimorphism. In the young females only, the Cre^-^ mice showed significantly higher marrow colony numbers when cultured with 4-OH tamoxifen compared with ethanol (Fig 3B: p<0.05), but not in the bone.

## Discussion

The negative effects of activated CreER^T2^ recombinase on colony forming abilities are seen here in both bone and marrow, suggesting it has wide spread relevance, potentially affecting other tissues. It is of note that there are a higher number of colonies forming in the bone CFU-F assays compared to marrow, which is consistent with previous studies [5,27]. The trabecular bone is an enriched source of mesenchymal progenitors which corroborates with higher numbers of bone colonies [3,28].

Here we show that the dramatic effect on the clonogenic ability is due to activated CreER^T2^ recombinase; a worrying finding for the scientific community as a whole. This raises questions as to whether activated CreER^T2^ recombinase is the cause of some phenotypes seen in published studies also using this construct. Very few studies control for CreER^T2^ toxicity effects with respect to such colony forming assays and may be wrongly accrediting their findings to a changed gene expression status.

It is interesting to speculate why activating CreER^T2^ recombinase is creating this phenotype *in vitro* and there are two potential explanations: Firstly, the CreER^T2^ recombinase protein is toxic. Secondly, the CreER^T2^ recombinase is acting on endogenous pseudo-*loxP* sites and is causing off target effects. It is possible that the CreER^T2^ recombinase enzyme is cleaving the DNA at endogenous pseudo *lox* sites which occur naturally in the genome and share some homology with *loxP* sites [29].

Cells expressing CreER^T2^ recombinase and lacking *loxP* sites undergo cell cycle arrest at the G2/M phase inhibiting cell growth and have chromosomal aberrations which lead to genetic instability [22,30,31]. The use of Cre in the generation of knock-in animals means that phenotypes associated with the knock-in allele may also be due, in part, to Cre-mediated mutations [31].

Mouse embryo fibroblasts with the CreER^T2^ knock-in allele driven by the endogenous ROSA26 promoter, similar to the CAGG driven CreER^T2^ used in this study, were investigated for toxicity effects [22]. These cells, which also lack *loxP* sites, were cultured with 4-OH tamoxifen resulting in a severe reduction of proliferation rates causing inhibited growth [22]. These data concur with the lack of colony growth seen by the CAGG-CreER^T2^ positive cells in the present study, suggesting a toxic effect.

Young female CreER^T2^ positive marrow cells formed significantly more colonies than CreER^T2^ negative cells. These cells were cultured in media with control ethanol, meaning the CreER^T2^ is not dissociated and therefore inactivate, suggesting that the mere presence of the CreER^T2^ construct is the only difference between these cells. This was only observed in the young female bone marrow cells, not adult.

It has been shown that CreER^T2^ can be leaky by still exerting an effect in the nucleus without the presence of 4-OH tamoxifen as well as via spontaneous *loxP* site recombination due to tamoxifen contamination between animals *in vivo* [32,33]. However, in this study CreER^T2^ positive cells plus 4-OH tamoxifen results in a reduction of colonies, whereas the opposite is seen with the vehicle control ethanol dismissing a leaking effect. Female marrow cells are the only case in which the negative CreER^T2^ and tamoxifen effect is not seen to be significant. This may possibly be due to the positive effect that CreER^T2^ alone seems to be having in these cells and therefore masking any negative effects.

Various gender differences are found within stem cell groups, including MSCs [13]. Osteogenic properties of MSCs are greater in cells cultured with 17β-estradiol, which show increased BMP, osteocalcin, calcium deposits, plus *ALP*, *Collagen I*, and *TGFβ1* gene expression [13]. Tamoxifen has estrogen agonistic effects on human bone tissues increasing bone cell numbers, as well as the number of S phase cells [34]. Tamoxifen is also known to inhibit bone resorption and osteoclast formation in an estrogen receptor dependent manner [35].

These links between tamoxifen, the estrogen receptor, and bone growth make this gender dimorphism an interesting observation and concur nicely with our young female bone marrow CreER^T2^ negative cells producing higher colony numbers when cultured with 4-OH tamoxifen than with ethanol. Again, this was only observed in the young bone marrow cells and not in the adult. This may be due to the higher number of stem cells in younger mice, compared to mature adults when the bone has finished growing [15].

In summary, this study demonstrates that, at least for colony forming capabilities, marrow and bone cells are affected by activated CreER^T2^ recombinase, rather than the deletion of a target gene. This raises concerns as to whether activated CreER^T2^ recombinase is the cause of other phenotypes seen in published studies using this construct. It also seems that the inactivated CreER^T2^ recombinase construct as well as gender and 4-OH tamoxifen are having effects on CFU-F assay outcomes.

In conclusion, these observations determine use of the Cre-*loxP* system inadvisable in combination with CFU-F assays. This study should also act as a warning to ensure appropriate controls are in place in order to extend the use of the Cre-*loxP* system in alternate assays.

## Experimental Procedures

### Animal models

Mice were sacrificed by Schedule One in this study. The work was performed under the appropriate Project License viewed by the Animal Welfare and Ethical Review Body (AWERB) of the University of Edinburgh and has been authorised by the Home Office in the United Kingdom.

CAGG-CreER^T2^ mice were obtained from Sue Monkley at Leicester University (http://www.informatics.jax.org/allele/key/7468?page=alleleDetail&key=7468). Mice were housed and bred in the University of Edinburgh/MRC IGMM animal facilities. All animal experiments were performed in accordance to approved personal and project Home Office licences and regulations.

### Isolation of murine bone marrow and bone mesenchymal progenitors

Mice were euthanized by cervical dislocation. Both the bilateral femur and tibia were dissected out. Each end of the long bones was removed and bone marrow flushed from the bone using DMEM media (containing 10% fetal calf serum (FCS), 1% penicillin/streptomycin, 0.5% glutamine and 0.5% sodium pyruvate) and a 25 gauge needle. Flushed marrow cells were dissociated using a 21 gauge needle.

To obtain the bone mesenchymal progenitors, pre-flushed long bones were crushed by pestle and mortar, and then digested with 3 mg/ml collagenase B (Roche) for 90 minutes at 37°C in constant motion. Cells were passed through a 70 micron cell strainer, washed and resuspended in DMEM media (containing 10% FCS, 1% penicillin/streptomycin, 0.5% glutamine and 0.5% sodium pyruvate).

### CFU-F assay

Three mice were used for each experiment, with three assays per mouse (i.e. technical triplicate). Adult CAGG-CreER^T2^ mice were 12–21 weeks old and young CAGG-CreER^T2^ mice were 4 weeks old. Cells were plated in 6-well culture plates at a density of 5x10^5^ cells in 2ml of MesenCult^®^ (StemCell Technologies) media per well. After 48 hours, adhered cells were washed with PBS, and then cultured in MesenCult^®^ media plus 1 μM 4-hydroxy(4-OH)tamoxifen (Sigma) or the same concentration of 100% ethanol vehicle (EtOH) for 72 hours. Colonies were then grown in MesenCult^®^ for 10 days before staining with 0.5% Cresyl Violet Acetate in methanol. Colonies of more than 1mm diameter were counted [36–38].

### Toluidine Blue Staining

Colonies were washed with pre-warmed PBS and fixed for 20 minutes with 10% neutral buffered formalin at room temperature. Colonies were stained in 0.1% toluidine blue (in 1% paraformaldehyde in PBS) for 1 hour, and then washed in distilled water.

### Alkaline Phosphatase Staining

Colonies were washed with PBS and fixed for 60 seconds with 10% neutral buffered formalin. Colonies were then washed with wash buffer (0.05% Tween 20 in PBS). Alkaline phosphatase activity was identified using BCIP/NBT substrate (1 BCIP/NBT tablet (Sigma) in 10 ml distilled water, stored in the dark). Colonies were incubated at room temperature in the dark for up to 10 minutes. Staining was checked every 2–3 minutes to assess progress. Cells stained blue-violet in the presence of alkaline phosphatase. Colonies were then washed with wash buffer and left in PBS.

### Genotyping PCR

Genomic DNA was extracted using FlexiGene DNA Kit (QIAGEN) and amplified with gene-specific primers (Sigma) with an annealing temperature of 58°C. Primers used for detecting Cre are: Cre/F (5’ gcattaccggtcgatgcaacgagtgatgag 3’) and Cre/R (5’ gagtgaacgaacctggtcgaaatcagtgcg 3’). DNA fragments were separated by electrophoresis on a 2% agarose gel to assess whether Cre was present or not. Cre presence shows a band at ~400 BP under UV illumination. The animals used in this study were heterozygotes, i.e. only contained one copy of the CreER recombinase construct.

### Statistical Analysis

Results are reported as mean ± standard error of the mean (SEM). The significance of two groups was analysed using the unpaired t test (p values are denoted by an asterisk. *p < 0.05; **p < 0.01; ***p < 0.001).

## Supporting Information

S1 Table A. Percentage of total colonies positive for ALP activity were not affected by CreER^T2^ presence, CreER^T2^ activation, ethanol presence, or 4-OH tamoxifen presence. Colonies were stained with ALP to assess the degree of bone formation. The mean percentage of total colonies (±SEM) which were ALP positive were not affected in male bone or male marrow cultures. [Table A: Normoxia Cre^+^ vs. Cre^-^ (with ethanol): p = 0.28, Hypoxia Cre^+^ vs. Cre^-^ (with ethanol): p = 0.32, Normoxia Cre^+^ vs. Cre^-^ (with tamoxifen): p = 0.61, Hypoxia Cre^+^ vs. Cre^-^ (with tamoxifen): p = 0.40. Normoxia Cre^-^ (ethanol) vs. Cre^-^ (tamoxifen): p = 0.75, Normoxia Cre^+^ (ethanol) vs. Cre^+^ (tamoxifen): p = 0.88, Hypoxia Cre^-^ (ethanol) vs. Cre^-^ (tamoxifen): p = 0.52, Normoxia Cre^+^ (ethanol) vs. Cre^+^ (tamoxifen): p = 0.53. Table B: Normoxia Cre^+^ vs. Cre^-^ (with ethanol): p = 0.37, Hypoxia Cre^+^ vs. Cre^-^ (with ethanol): p = 0.78, Normoxia Cre^+^ vs. Cre^-^ (with tamoxifen): p = 0.36, Hypoxia Cre^+^ vs. Cre^-^ (with tamoxifen): p = 0.35. Normoxia Cre^-^ (ethanol) vs. Cre^-^ (tamoxifen): p = 0.71, Normoxia Cre^+^ (ethanol) vs. Cre^+^ (tamoxifen): p = 0.61, Hypoxia Cre^-^ (ethanol) vs. Cre^-^ (tamoxifen): p = 0.88, Normoxia Cre^+^ (ethanol) vs. Cre^+^ (tamoxifen): p = 0.32.] (Cre^-^: n = 3 experiments. Cre^+^: n = 3 experiments. All experiments were performed in technical triplicate).
(DOCX)
