# Supplementary material for: Effects of CreERT2, 4-OH Tamoxifen, and Gender on CFU-F Assays
Source: PLoS One. 2016 Feb 1;11(2):e0148105. doi: 10.1371/journal.pone.0148105 (PMC4734617; doi:10.1371/journal.pone.0148105)
Supplement: S1 Table — Colonies were stained with ALP to assess the degree of bone formation. The mean percentage of total colonies (±SEM) which were ALP positive were not affected in male bone or male marrow cultures. [Table A: Normoxia Cre+ vs. Cre- (with ethanol): p = 0.28, Hypoxia Cre+ vs. Cre- (with ethanol): p = 0.32, Normoxia Cre+ vs. Cre- (with tamoxifen): p = 0.61, Hypoxia Cre+ vs. Cre- (with tamoxifen): p = 0.40. Normoxia Cre- (ethanol) vs. Cre- (tamoxifen): p = 0.75, Normoxia Cre+ (ethanol) vs. Cre+ (tamoxifen): p = 0.88, Hypoxia Cre- (ethanol) vs. Cre- (tamoxifen): p = 0.52, Normoxia Cre+ (ethanol) vs. Cre+ (tamoxifen): p = 0.53. Table B: Normoxia Cre+ vs. Cre- (with ethanol): p = 0.37, Hypoxia Cre+ vs. Cre- (with ethanol): p = 0.78, Normoxia Cre+ vs. Cre- (with tamoxifen): p = 0.36, Hypoxia Cre+ vs. Cre- (with tamoxifen): p = 0.35. Normoxia Cre- (ethanol) vs. Cre- (tamoxifen): p = 0.71, Normoxia Cre+ (ethanol) vs. Cre+ (tamoxifen): p = 0.61, Hypoxia Cre- (ethanol) vs. Cre- (tamoxifen): p = 0.88, Normoxia Cre+ (ethanol) vs. Cre+ (tamoxifen): p = 0.32.] (Cre-: n = 3 experiments. Cre+: n = 3 experiments. All experiments were performed in technical triplicate). (DOCX) [file pone.0148105.s001.docx]

|  | Normoxia |  | Hypoxia |  |
| --- | --- | --- | --- | --- |
|  | Cre- | Cre+ | Cre- | Cre+ |
| Cultured with Ethanol | 19.44 (±10.02) | 37.60 (±10.53) | 69.63 (±23.36) | 39.62 (±12.55) |
| Cultured with Tamoxifen | 23.64 (±6.95) | 34.28 (± 17.84) | 49.07 (±17.69) | 22.22 (±22.22) |

**S1 Table A. Percentage of male marrow colonies which are ALP positive.**

**S1 Table B. Percentage of male bone colonies which are ALP positive.**

|  | Normoxia |  | Hypoxia |  |
| --- | --- | --- | --- | --- |
|  | Cre- | Cre+ | Cre- | Cre+ |
| Cultured with Ethanol | 25.40 (±15.05) | 10.02 (±2.93) | 17.68 (±4.75) | 16.01 (±3.32) |
| Cultured with Tamoxifen | 18.19 (±9.83) | 6.25 (±6.25) | 16.52 (±5.14) | 7.14 (±7.14) |

**S1 Table. A. Percentage of total colonies positive for ALP activity were not affected by CreER^T2^ presence, CreER^T2^ activation, ethanol presence, or 4-OH tamoxifen presence.**

Colonies were stained with ALP to assess the degree of bone formation. The mean percentage of total colonies (±SEM) which were ALP positive were not affected in male bone or male marrow cultures.

[Table A: Normoxia Cre^+^ vs. Cre^-^ (with ethanol): p=0.28, Hypoxia Cre^+^ vs. Cre^-^ (with ethanol): p=0.32, Normoxia Cre^+^ vs. Cre^-^ (with tamoxifen): p=0.61, Hypoxia Cre^+^ vs. Cre^-^ (with tamoxifen): p=0.40. Normoxia Cre^-^ (ethanol) vs. Cre^-^ (tamoxifen): p=0.75, Normoxia Cre^+^ (ethanol) vs. Cre^+^ (tamoxifen): p=0.88, Hypoxia Cre^-^ (ethanol) vs. Cre^-^ (tamoxifen): p=0.52, Normoxia Cre^+^ (ethanol) vs. Cre^+^ (tamoxifen): p=0.53.

Table B: Normoxia Cre^+^ vs. Cre^-^ (with ethanol): p=0.37, Hypoxia Cre^+^ vs. Cre^-^ (with ethanol): p=0.78, Normoxia Cre^+^ vs. Cre^-^ (with tamoxifen): p=0.36, Hypoxia Cre^+^ vs. Cre^-^ (with tamoxifen): p=0.35. Normoxia Cre^-^ (ethanol) vs. Cre^-^ (tamoxifen): p=0.71, Normoxia Cre^+^ (ethanol) vs. Cre^+^ (tamoxifen): p=0.61, Hypoxia Cre^-^ (ethanol) vs. Cre^-^ (tamoxifen): p=0.88, Normoxia Cre^+^ (ethanol) vs. Cre^+^ (tamoxifen): p=0.32]

(Cre^-^: n=3 experiments. Cre^+^: n=3 experiments. All experiments were performed in technical triplicate)
